# The Coronavirus Double Threat: A Rare Presentation of Chest Pain in a Young Female

**DOI:** 10.7759/cureus.37274

**Published:** 2023-04-07

**Authors:** Hanad Bashir, Daniya Muhammad Haroon, Gauranga Mahalwar, Ankur Kalra, Adeeb Alquthami

**Affiliations:** 1 Cardiovascular Medicine, The Christ Hospital, Cincinnati, USA; 2 Internal Medicine, Bahria University Medical and Dental College, Karachi, PAK; 3 Internal Medicine, Cleveland Clinic Akron General, Akron, USA; 4 Vascular and Thoracic Department, Cleveland Clinic Akron General, Akron, USA; 5 Cardiology, Cleveland Clinic Akron General, Akron, USA

**Keywords:** coronary angiography, transient ischemic attack, left ventricular thrombus, spontaneous coronary artery dissection, scad, covid-19, acute coronary syndrome

## Abstract

Severe acute respiratory syndrome coronavirus 2 (SARS-COV 2) led to global coronavirus disease 2019 (COVID-19) pandemic. The virus affects the respiratory system predominantly and has resulted in multiorgan complications. Myocarditis, acute coronary syndrome (ACS), cardiogenic shock, and sudden cardiac death were common cardiac manifestations of COVID-19. Spontaneous coronary artery dissection (SCAD) is a rare form of coronary artery disease that is previously reported in patients with COVID-19. SCAD usually occurs in a middle-aged woman with few or without any cardiovascular risk factors. The gold standard for its diagnosis is coronary angiography. The SCAD treatment recommendations depend on the hemodynamic status: conservative therapy in hemodynamically stable SCAD patients and urgent revascularization in hemodynamically unstable SCAD patients. The exact pathophysiology of COVID-19 associated with SCAD is unknown. It is considered a combination of systemic inflammatory response and localized vascular inflammation. The case reported is of COVID-19-associated SCAD in a patient with no history of cardiovascular disease later complicated by the transient ischemic attack (TIA) and left ventricular (LV) thrombus.

## Introduction

The coronavirus disease 2019 (COVID-19) pandemic, caused by the severe acute respiratory syndrome coronavirus 2 (SARS-CoV-2), has significantly impacted public health on a global scale. It was first reported in Wuhan, China, in December 2019 and has since spread globally, infecting over 651 million people and causing over 6.80 million deaths as of March 2023 [1,2]. The cardiovascular presentation of SARS-CoV-2 is variable and can pose a challenge concerning diagnosis and management. Here, we present a case report of spontaneous coronary artery dissection (SCAD) accompanied by left ventricular (LV) thrombus formation and discuss the diagnostic algorithm and management guidelines.

## Case presentation

**Figure 1:**
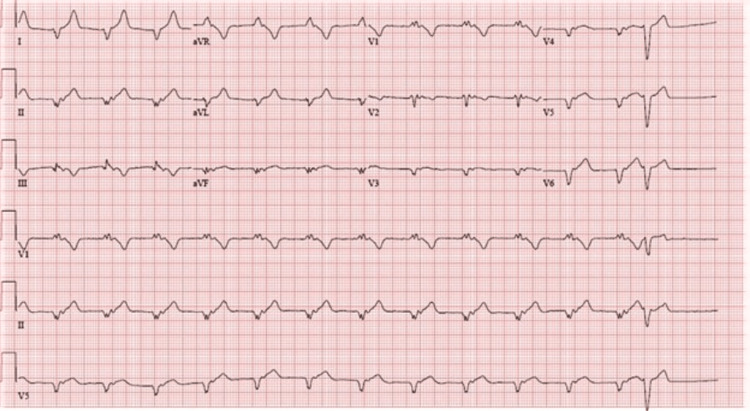
Electrocardiogram

A 36-year-old female with a past medical history of morbid obesity with a body mass index of 49 kg/m^2^ presented to the emergency department with complaints of a one-day history of severe chest pain that started a few hours before presentation. The chest pain was described as substernal eight out of 10 severity with radiation to her neck and arms bilaterally. Aspirin 324 mg and sublingual nitroglycerin were given to her and minimally improved her symptoms. On physical examination, she appeared anxious, however cooperative, with a temperature of 37.8°C, a heart rate of 84 beats/minute, blood pressure of 119/66 mm Hg, a respiratory rate of 11 breaths per minute, and pulse oximetry of 96% on room air. Her heart and lung sounds were normal. Her laboratory results were notable for an initial TNT high-sensitive troponin of 8 (<12 ng/L) increased to 237 (<12 ng/L). Her initial electrocardiogram on presentation showed a right bundle branch block with hyperacute T waves (Figure 1).

**Figure 2:**
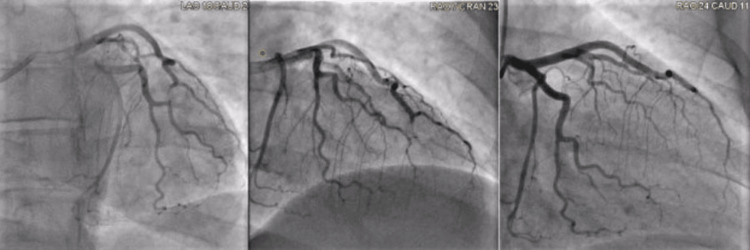
Coronary angiography

The patient subsequently started on heparin infusion and also tested positive for SARS-CoV-2 on a PCR nasal swab. Since the patient continued to have chest pain despite multiple doses of nitroglycerin, a left heart catheterization was performed. A pre-flushed six-French sheath was inserted into the radial artery via the Seldinger technique. Left anterior descending (LAD) showed total occlusion distal portion with thrombolysis in myocardial infarction (TIMI) flow of zero. The left heart catheterization revealed likely SCAD (Figure 2).

**Figure 3:**
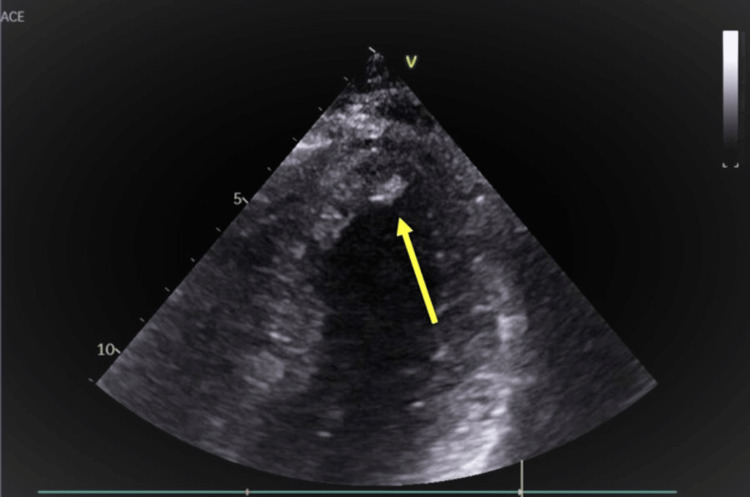
Left ventricular thrombus

The left ventriculogram showed a dilated left ventricle with moderate LV systolic dysfunction. The LV ejection fraction was 35%. She was subsequently started on aspirin, atorvastatin, ticagrelor, and metoprolol tartrate and got discharged with plans for a cardiology follow-up in a month. She did not have any features of connective tissue disease like hypertelorism, high-arched palate, and varicose veins. Echocardiogram got deferred due to active COVID-19 infection according to institutional guidelines and the fact that she had an assessment of her LV systolic function on the ventriculogram. Two days post-discharge, the patient presented again with symptoms of a sudden loss of peripheral vision on her right side with associated dizziness. She underwent a CT of her head and neck that did not show any acute abnormalities. MRI of the brain also revealed no evidence of an acute ischemic infarct. CT angiography with contrast revealed a moderately dilated left and right atrium and patent foramen ovale. LV apical thrombus was also seen. An echocardiogram revealed a fixed mural LV thrombosis in the apex measuring 2.7 cm x 1.7 cm x 0.9 cm (Figure 3).

**Figure 4:**
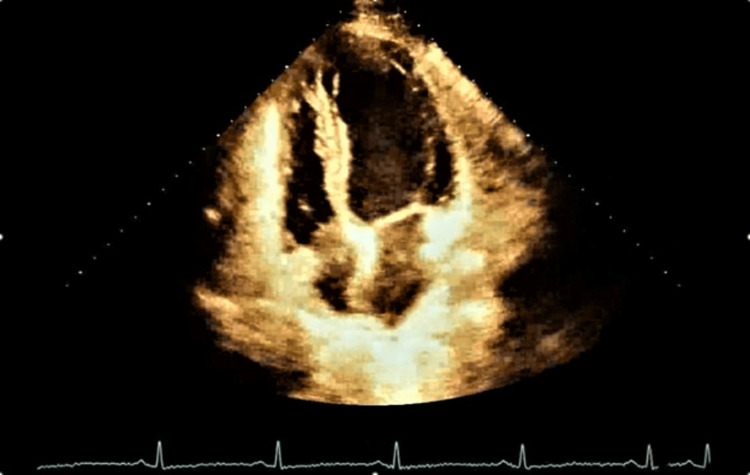
Resolved left ventricular thrombus

She was subsequently started on a heparin drip and then transitioned to warfarin. She was discharged with plans for an outpatient echocardiogram, cardiac magnetic resonance, carotid and renal artery Doppler ultrasound, and genetic testing once the patient's PCR of COVID-19 turns negative. Three months later, the patient had a repeat echocardiogram that showed resolution of her LV thrombus and improvement of her LV ejection fraction to 60% (Figure 4).

## Discussion

The COVID-19 caused by the SARS-CoV-2 virus has affected millions of people around the globe. Although it primarily affects the pulmonary system, the virus also involves other organ systems, including the cardiovascular system. The most commonly reported cardiac manifestations of COVID-19 include myocarditis, acute coronary syndrome (ACS), cardiogenic shock, and even sudden cardiac death [3]. SCAD, an uncommon manifestation of coronary artery disease, has been previously reported in patients with COVID-19. Per Cosma et al., 11 cases of SCAD associated with COVID-19 infection have been reported so far [4]. Our case presents a unique challenge of treating LV thrombus with SCAD in a patient with COVID-19 infection. The etiology of SCAD differs from atherosclerotic coronary artery disease (ASCAD) by the absence of traditional cardiovascular risk factors and stressors associated with ASCAD. Although other factors such as smoking, hypertension, hyperlipidemia, and obesity can contribute to SCAD and can result in ACS, respiratory viral infections, such as influenza, have also been associated with cases of ACS, which may be due to arterial and coronary inflammation [5,6]. The hypothesis that SARS-CoV-2 infection causes T-cell infiltration of the adventitia and periadventitial fat results in increased cytokine and protease production (local or systemic) that results in the possibility of plaque rupture and subsequent dissection [7]. Another proposed mechanism for SCAD in COVID-19 infection is that SARS-CoV-2 increases angiogenesis, which results in the proliferation of arterial vasa vasorum. Newly formed vasa vasorum are more prone to inflammatory cell invasion of the tunica media and adventitia, increasing the risk of SCAD [8]. The definitive diagnosis requires coronary angiography. In addition, imaging, such as left ventriculography and echocardiography, aids in establishing the diagnosis. Management involves the administration of intracoronary nitrates, intracoronary imaging, when safe and available, and/or follow-up noninvasive or invasive coronary imaging may help distinguish SCAD from other etiologies [9]. Although angiographic diagnosis is doable in SCAD cases mostly and conservative management is preferred, intracoronary imaging applies for uncertain diagnosis or where percutaneous coronary intervention (PCI) is required. However, the significant complication of PCI for SCAD is stent embolization, which along with other complications should be kept in mind [10]. Current management of SCAD focuses on conservative measures to restore TIMI flow grade 3 in contrast to the approach in atherosclerotic ACS [9]. Post-procedural management should focus on relieving symptoms with anti-anginal and antihypertensives and identifying patients with evidence of progressive ischemia where invasive reinvestigation is mandatory [9]. Patients who undergo PCI should receive standard guideline-based dual antiplatelet therapy (DAPT) [11]. Most experts recommend aspirin use for at least one year and frequently after SCAD in patients who receive medical treatment with no contraindications, but the overall consensus on the use of antiplatelet therapy is still not well established [12]. Similarly, anticoagulation must weigh the risk-benefit of a thrombus reduction burden versus an extension of the intramural hemorrhage. Outside of alternative indications for anticoagulation, once the SCAD diagnosis establishes, anticoagulation is recommended to be stopped [13]. The LV thrombus formation in our patient attributes to multiple factors. These include LV regional wall akinesia and dyskinesia resulting in blood stasis and prolonged ischemia leading to subendocardial tissue injury with inflammatory changes. COVID-19 infection in and of itself has been known to contribute to a hypercoagulable state and profounds pro-inflammatory state as evidenced by high C-reactive protein, lactate dehydrogenase, ferritin, interleukin-6, and D-dimer levels along with the aforementioned factors [14]. The presence of antiphospholipid antibody syndrome also contributed to thrombosis formation in our patient. Our case is unique as the patient had both coronary dissections and LV thrombus, which presented a unique therapeutic challenge. One case report mentioned a large LV thrombus in a patient with COVID-19 pneumonia [15].

## Conclusions

We describe how a patient with COVID-19 infection was found to have SCAD complicated by a transient ischemic attack (TIA) and LV thrombus. After weighing the benefits and risks of anticoagulation in the acute phase of SCAD, she was started on warfarin for anticoagulation with clopidogrel. A follow-up echocardiogram three months later showed the resolution of the LV thrombus with no additional coronary-related symptoms. Our case highlights the successful treatment of complicated SCAD-related LV thrombus in the setting of COVID-19 infection. 
